# Oral susceptibility to ivermectin is over fifty times greater in a wild population of *Anopheles albimanus* mosquitoes from Belize than the STECLA laboratory reference strain of this mosquito

**DOI:** 10.1186/s12936-022-04092-y

**Published:** 2022-03-04

**Authors:** Staci M. Dreyer, Kelsey J. Morin, Marla Magaña, Marie Pott, Donovan Leiva, Nicole L. Achee, John P. Grieco, Jefferson A. Vaughan

**Affiliations:** 1 University of North Dakota, Grand Forks, ND 58202 USA; 2 Belize Vector and Ecology Center, Orange Walk Town, Belize; 3 University of Notre Dame, South Bend, IN 46556 USA

**Keywords:** *Anopheles albimanus*, Ivermectin, STECLA, Belize

## Abstract

**Background:**

The STECLA strain of *Anopheles albimanus* has been in continuous colony for many years and is the reference strain on which genomic studies for the species are based. Recently, the STECLA strain was demonstrated to be much less susceptible to ivermectin ingested in a blood meal (4-day LC_50_ of 1468 ng/ml) than all other *Anopheles* species tested to-date (LC_50_ values range from 7 to 56 ng/ml). The ability of *An. albimanus* to survive ingestion of ivermectin at concentrations far beyond that typically found in the blood of ivermectin-treated people or livestock (i.e., 30–70 ng/ml) could invalidate the use of ivermectin as a malaria vector control strategy in areas where *An. albimanus* is a primary vector.

**Methods:**

To investigate this, host-seeking *An. albimanus* were captured in northern Belize and used in membrane feeding bioassays of ivermectin, employing the same methods as described earlier with the STECLA strain.

**Results:**

Field-collected *An. albimanus* in Belize were 55 times more susceptible to ingested ivermectin than were the STECLA reference strain. Oral susceptibility to ivermectin in wild *An. albimanus* from Belize (4-day LC_50_ of 26 ng/ml) was equivalent to that of other *Anopheles* species tested.

**Conclusions:**

Contrary to initial assessments using a highly inbred strain of mosquito, laboratory studies using a field population indicate that ivermectin treatment of livestock could reduce *An. albimanus* populations in areas of Central America and the Caribbean where malaria transmission may occur. Toxicity screening of ivermectin and other systemic parasiticides for malaria control should examine wild populations of the vector species being targeted.

## Background

Ivermectin has long been an important drug for treating livestock against parasitic nematodes and arthropods (e.g., ticks) and more recently, for treating humans against filarial nematodes that cause lymphatic filariasis and onchocerciasis. Ivermectin has potential importance in the global effort to eliminate malaria because of its ability to reduce malaria vector populations [1]. When ingested by *Anopheles* mosquitoes at concentrations normally found in the plasma of treated people or livestock, ivermectin has been shown to reduce the survivorship and fecundity of almost every *Anopheles* species in which the drug has been tested [2–15]. The one exception has been the Central American vector, *Anopheles albimanus*.

In recent laboratory studies [16], it was reported that the concentration required to kill 50% (i.e., the LC_50_) of *An. albimanus* (LC_50_ = 1468 ng/ml) was so much higher than the maximum concentration of ivermectin typically found in the sera of treated humans or cattle (i.e., 30–70 ng/ml [1, 17–19]) that ivermectin would be useless as a malaria control strategy against this mosquito species. The following year a pilot trial was conducted with cattle in northern Belize [20]. One of the animals was injected with a commercial formulation of ivermectin (Labimectin®, LabiPharma, Guatemala City, GUATEMALA) following the instructions on the label. This treatment was to serve as an extra ‘negative control’. Unexpectedly, wild *An. albimanus* that fed on the ivermectin-injected animal experienced significantly higher mortality than did wild *An. albimanus* fed on untreated cattle. It appeared that wild *An. albimanus* mosquitoes from northern Belize were more susceptible to ingested ivermectin than were the STECLA laboratory strain of *An. albimanus* mosquitoes obtained from BEI Resources (Manassas, VA USA). The STECLA strain is the *An. albimanus* reference strain used for many studies, including a recent physical genome map [21]. This report documents the acute oral susceptibility to ivermectin of wild-caught *An. albimanus* in Belize (hereafter referred as *An. albimanus* BELIZE) and compares it with that of *An. albimanus* STECLA, as well as with other *Anopheles* species that have been similarly tested.

## Methods

### Mosquitoes

Host-seeking mosquitoes were collected during nighttime human landing catches in San Roman Rio Hondo, Orange Walk District, Belize. Mosquitoes were transported to the Belize Vector and Ecology Center laboratory in Orange Walk Town, Belize. *Anopheles albimanus* was distinguished from other anopheline species based on the characteristic banding pattern on the hind tarsi [22]. After identification, *An. albimanus* BELIZE were transferred into smaller (ca. 0.5 L) cylindrical plastic cages with mesh tops at a density of 15–30 mosquitoes. Mosquitoes were maintained at 26 °C with access to 8% honey solution ad libitum.

### Membrane feeding

Stock solutions of ivermectin (Product No. 18898, Sigma-Aldrich, St. Louis MO, USA) at a concentration of 2 mg ivermectin per 1 ml dimethyl sulfoxide were prepared at the University of North Dakota, frozen, and transported by air to Belize City and by automobile to Belize Vector and Ecology Center, Orange Walk Town, Belize (approximately a one-hour drive). Stock solutions were diluted in water to make initial starting concentrations. Final ivermectin concentrations (i.e., 10, 25, 50, 150, 300, 1000 ng/ml) were then prepared by adding appropriate volumes of human blood to a final volume of 8 ml. The control group received blood with no additives. Blood mixtures were kept warm prior to feeding. Natural ham collagen, pre-soaked in distilled water, was used as the material through which mosquitoes probed and fed. The collagen was affixed to glass membrane feeders with rubber bands, feeders were connected to one another with rubber tubing, and warm water (37 °C) was circulated through the feeders. Membrane feeders were then placed on individual cages containing 15 to 30 wild-caught mosquitoes and the pre-warmed blood mixtures were pipetted into the feeders. Mosquitoes were allowed 90 min to feed in darkness. Afterwards, unfed mosquitoes were removed. Engorged mosquitoes were maintained at 26 °C with access to 8% honey solution ad libitum. Cages were checked every day and dead mosquitoes were counted and removed. After four days, surviving mosquitoes were counted and trial runs were terminated at that point. As much as possible, the methodologies used in this trial were consistent with that used by Dreyer et al. [16] with the notable exceptions that in this trial, the initial dilutions of ivermectin stock solution were prepared using water instead of phosphate buffered saline, and human blood (rather than cow blood) was used in this trial for the final mixtures fed to mosquitoes.

### Data analysis

Mosquito mortalities observed within experimental groups were adjusted for mortality that occurred within corresponding control groups using Abbott’s formula [23]. Only experimental trials having control mortalities less than 20% were used for further data analyses. Log-probit analyses were conducted on the corrected percent moralities to estimate LC_50_ values (Minitab Inc., State College PA, USA). Mosquito survivorship was analysed with a Kaplan–Meier survival analysis and Log-rank Mantel-Cox test (GraphPad Software, La Jolla CA USA). A p-value of less than or equal to 0.05 was used throughout to indicate statistical difference between experimental groups.

## Results

**Fig. 1:**
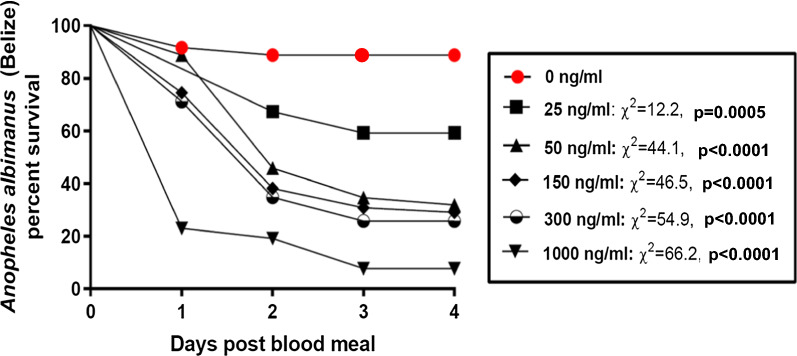
Kaplan–Meier daily proportion of surviving *Anopheles albimanus* BELIZE after ingesting ivermectin at various concentrations

A total 352 fully engorged mosquitoes over five separate feeding trials were used to determine the acute oral toxicity of ivermectin for *An. albimanus* BELIZE, collected in the field from northern Belize. The estimated average membrane-feeding rate was 31.5%. Post-feeding mosquito mortality was protracted and occurred over a period of several days after ingestion of treated blood (Fig. 1), as reported for other *Anopheles* species ingesting ivermectin. The LC_50_ (lower and upper 95% confidence intervals) at day 4 post-feeding was 26.4 ng/ml (13.7–51.0); over 55-fold higher than that reported for the STECLA laboratory reference strain of *An. albimanus* (LC_50_ = 1468 ng/ml) using the same methodologies [16].

## Discussion

With the notable exception of the *An. albimanus* STECLA, all *Anopheles* species tested thus far with ivermectin using membrane-feeding techniques, have LC_50_ values (i.e., 7 to 56 ng/ml, Table 1) well within the typical peak plasma concentrations of ivermectin reported for humans and livestock (e.g., 30–70 ng/ml) following standard drug administration at approved doses. Thus, all *Anopheles* species examined to date are theoretically susceptible to population reduction *via* targeted administration of ivermectin to humans and livestock. This is the first study to quantify oral susceptibility to ivermectin in a field population of *Anopheles* using the membrane feeding bioassay technique. Previous studies using this standardized technique have relied on laboratory strains of mosquitoes that have been in continuous colony for many years. Not surprisingly, there was more heterogeneity in the response to ingested ivermectin with the Belize field population, as indicated by wider confidence intervals around the LC_50_ value than observed in colonized mosquitoes (Table 1). Similarly, there was a flatter slope in the dose-response curve of wild *An. albimanus* BELIZE than observed for the STECLA strain of *An. albimanus* and for laboratory strains of *Anopheles stephensi* STE2 and *Anopheles arabiensis* DONGOLA (Table 2). Greater heterogeneity in the response to ivermectin by a wild population may have resulted from several sources, including; smaller sample sizes examined, testing mosquitoes of unknown age and physiological condition, and to the greater overall genetic diversity inherent in field populations versus inbred laboratory strains. Importantly, the findings that different populations of *An. albimanus* (BELIZE versus STECLA) vary widely in their susceptibilities to ivermectin and that the response to ivermectin in a wild population is more heterogenous than in laboratory populations suggest that the development of ivermectin-resistant populations of *An. albimanus* in nature is possible. That possibility is underscored by the fact that in our trials, two of 26 (7.7%) *An. albimanus* BELIZE mosquitoes that ingested 1000 ng/ml of ivermectin were able to survive the 4-day post-feeding interval.

**Table 1:** Acute oral toxicities to ivermectin for *Anopheles* species using *in vitro* membrane feeding techniques, ranked according to susceptibility

*Anopheles* species	Mosquito strain and history*	Mortality assessment period (day)	N	Oral LC_50_ (95% CL)	References
*stephensi*	STE2; Long-standing	4	573	7 (5, 9)	[16]
*arabiensis*	DONGOLA; Long-standing	9	515	8 (6, 10)	[7]
*minimus*	AFRIMS; Long-standing	7	2376	16 (12, 19)	[13]
*gambiae s.s.*	KISUMU; Long-standing	9	Not reported	20 ± 3	[2]
*gambiae s.s.*	G3; Long-standing	5	2013	22 (18, 27)	[5]
*albimanus*	BELIZE; Field-collected	4	352	26 (14, 51)	Present study
*campestris*	AFRIMS; Long-standing	7	2786	26 (22, 30)	[13]
*sawadwongporni*	AFRIMS; Long-standing	7	1446	27 (25, 29)	[13]
*darlingi*	NAMRU-6; Recent	7	6161	43 (37, 49)	[14]
*aquasalis*	FMT-HVD; Long-standing	5	1415	47 (45, 49)	[11]
*dirus*	AFRIMS; Long-standing	7	5029	56 (52, 59)	[13]
*albimanus*	STECLA; Long-standing	4	582	1468 (1153, 1965)	[16]

* ‘Long-standing’ is defined as more than 5 years of continuous colony prior to testing. ‘Recent’ is defined as two to three years in colony prior to testing

**Table 2:** Regression parameters describing the dose-response of various *Anopheles* species and strains to ingested ivermectin

Species/Strain	LC_50_	N	df	Intercept	Slope	References
*An. albimanus* BELIZE	26.4	352	5	− 1.1	0.78	Present study
*An. albimanus* STECLA	1468.0	582	5	− 4.5	1.41	[16]
*An. stephensi* STE2	7.0	573	5	− 1.2	1.37	[16]
*An. arabiensis* DONGOLA	7.9	518	5	− 2.2	1.06	[7]

## Conclusions

This study illustrates the importance of including wild-caught indigenous populations of vectors (as opposed to sole reliance on laboratory strains) during *in vitro* toxicological screening of ivermectin and other systemic parasiticides. By screening wild populations of a targeted vector species, investigators may know better what to expect in field trials that involve treating entire herds of livestock.

## Data Availability

The data analysed during this study are available on request from the corresponding author.

## Abbreviation

LC_50_: Concentration of ivermectin required to kill 50% of treated mosquitoes
